# Multiparametric magnetic resonance imaging in the assessment of anti-EGFRvIII chimeric antigen receptor T cell therapy in patients with recurrent glioblastoma

**DOI:** 10.1038/s41416-018-0342-0

**Published:** 2018-11-27

**Authors:** Sumei Wang, Donald M. O’Rourke, Sanjeev Chawla, Gaurav Verma, MacLean P. Nasrallah, Jennifer J. D. Morrissette, Gabriela Plesa, Carl H. June, Steven Brem, Eileen Maloney, Arati Desai, Ronald L. Wolf, Harish Poptani, Suyash Mohan

**Affiliations:** 1 Department of Radiology, Division of Neuroradiology, Perelman School of Medicine at the University of Pennsylvania, Philadelphia, PA USA; 2 Department of Neurosurgery, Perelman School of Medicine at the University of Pennsylvania, Philadelphia, PA USA; 3 Department of Pathology and Laboratory Medicine, Division of Neuropathology, Perelman School of Medicine at the University of Pennsylvania, Philadelphia, PA USA; 4 Department of Pathology and Laboratory Medicine, Division of Precision and Computational Diagnostics, Perelman School of Medicine at the University of Pennsylvania, Philadelphia, PA USA; 5 Center for Cellular Immunotherapies, University of Pennsylvania, Philadelphia, PA USA; 6 Department of Medicine, Division of Hematology-Oncology, Perelman School of Medicine at the University of Pennsylvania, Philadelphia, PA USA; 7 Department of Cellular and Molecular Physiology, University of Liverpool, Liverpool, UK

**Keywords:** Cancer imaging, Oncology

## Abstract

EGFRvIII targeted chimeric antigen receptor T (CAR-T) cell therapy has recently been reported for treating glioblastomas (GBMs); however, physiology-based MRI parameters have not been evaluated in this setting. Ten patients underwent multiparametric MRI at baseline, 1, 2 and 3 months after CAR-T therapy. Logistic regression model derived progression probabilities (PP) using imaging parameters were used to assess treatment response. Four lesions from “early surgery” group demonstrated high PP at baseline suggestive of progression, which was confirmed histologically. Out of eight lesions from remaining six patients, three lesions with low PP at baseline remained stable. Two lesions with high PP at baseline were associated with large decreases in PP reflecting treatment response, whereas other two lesions with high PP at baseline continued to demonstrate progression. One patient didn’t have baseline data but demonstrated progression on follow-up. Our findings indicate that multiparametric MRI may be helpful in monitoring CAR-T related early therapeutic changes in GBM patients.

## Introduction

Glioblastoma (GBM) is the most common primary malignant brain tumor in adults with poor prognosis. Recurrence is almost inevitable and the median survival for these recurrent patients is only 6.6–9.6 months.^1^ Epidermal growth factor receptor variant III (EGFRvIII) is expressed in about one third of GBM patients, promotes oncogenesis and is associated with poor prognosis.^2,3^ A recent study demonstrated successful synthesis, delivery, and acceptable safety profile of chimeric antigen receptor T (CAR-T) cell therapy targeting against EGFRvIII epitope in patients with recurrent GBM.^4^ Since immunotherapy including CAR-T therapy, triggers patient’s immune system to fight cancer cells, a pronounced inflammatory response occurs within the tumour bed,^5^ complicating the appearance on conventional MRI for evaluation of therapeutic response. Multiparametric analysis using diffusion tensor imaging (DTI),^6^ dynamic susceptibility contrast (DSC) perfusion imaging^7^ and proton MR spectroscopy^8^ have been reported to distinguish true progression (TP) from pseudo-progression (PsP) with high accuracy.^6,9^ This rationale formed the basis of this study in which treatment response of CART-EGFRvIII immunotherapy in recurrent GBMs was evaluated using DTI, DSC and spectroscopic imaging.

## Methods

The study was approved by the Institutional Review Board. Informed consent was obtained from all patients. Ten recurrent GBM patients (5M/5F, mean age, 60.56 ± 10.31 years) were included based on inclusion criteria (Supplementary Material). Clinical/demographic information, EGFRvIII expression levels and overall survival (OS) were described in Table S1. Baseline images were acquired within one week before CAR-T cell infusion. Tumour progression was determined based on a combination of clinical status and RANO criteria.

Seven of 10 patients underwent resection after demonstrating progression following CAR-T cell infusion. Patients were divided into three groups according to the time of repeat surgery after CAR-T cell infusion: (1) no surgery (*no surgery group*, *n* = 3); (2) surgery within a month (*early surgery group*, *n* = 4); (3) surgery over a month (*late surgery group*, *n* = 3) (Table S1).

Data acquisition/analysis of DTI, DSC and 3-D echo planar spectroscopic imaging (3D-EPSI) sequences were performed as previously described.^6,8,10^ All contrast enhancing lesions (*n* = 12) ≥ 1 cm^3^ were selected for quantitative analysis. A semi-automatic segmentation approach was used to generate a mask from the enhancing region of the neoplasm (Supplementary Material). The enhancing and central non-enhancing regions were used to compute tumour volume. Mean diffusivity (MD), fractional anisotropy (FA), linear anisotropy (CL), planar anisotropy (CP), spherical anisotropy (CS), relative cerebral blood volume (rCBV) and choline/creatine (Cho/Cr) ratio from enhancing lesions were estimated at each time point. Percent changes for each parameter between baseline and subsequent scans (N) were calculated as (N – baseline)/baseline × 100 for the non-surgery and late surgery groups. Baseline 3D-EPSI data were available from two lesions in one patient. Hence, percentage changes in Cho/Cr from these two lesions were also evaluated.

In our previous study,^6^ for patients who underwent surgery and chemoradiation therapy (CRT) and exhibited new enhancing lesions on follow-up imaging within six months, a combination of FA, CL and rCBV_max_ was reported to be the best model in differentiating PsP from TP with high accuracy (AUC 0.91). This model was determined based on the histological analysis of surgical samples. Therefore, these three parameters were used in this study to compute the progression probabilities (PP) of tumour progression of each lesion at each time point using the following regression equation^6^:

$$f({\mathrm{FA}},{\mathrm{CL}},{\mathrm{rCBV}}_{\mathrm{max}}) = \frac{1}{{1 + \exp ( - (\beta_0 + \beta_ 1{\mathrm{FA}} + \beta_2{\mathrm{CL}} + \beta_3{\mathrm{rCBV}_{\mathrm{max}}}))}}$$

where *β*_0_ = −16.17, *β*_1_ = 194.01, *β*_2_ = −285.65, and *β*_3_ = 1.21. Lesions were considered TP (predominant viable tumour) if the predictive PP was ≥ 50% and PsP (predominant treatment effects) if predictive PP was ≤ 50%.

In order to evaluate CAR-T treatment efficacy, we included 10 recurrent GBM patients without CAR-T therapy (Table S2) and calculated PP values.

OS was measured from the date of diagnosis and CAR-T cell infusion to the date of death for deceased patients, or the date of last clinical follow-up for surviving patients.

## Results

Three out of 10 patients died within 6 months after CAR-T cell infusion. Six patients survived > 6 months before succumbing to the disease. One patient (209) was still alive at the time of the writing of this manuscript with a survival of 34.0 months (1033 days). Median OS from all 10 patients was 247 days (Table S1).

**Fig. 1:**
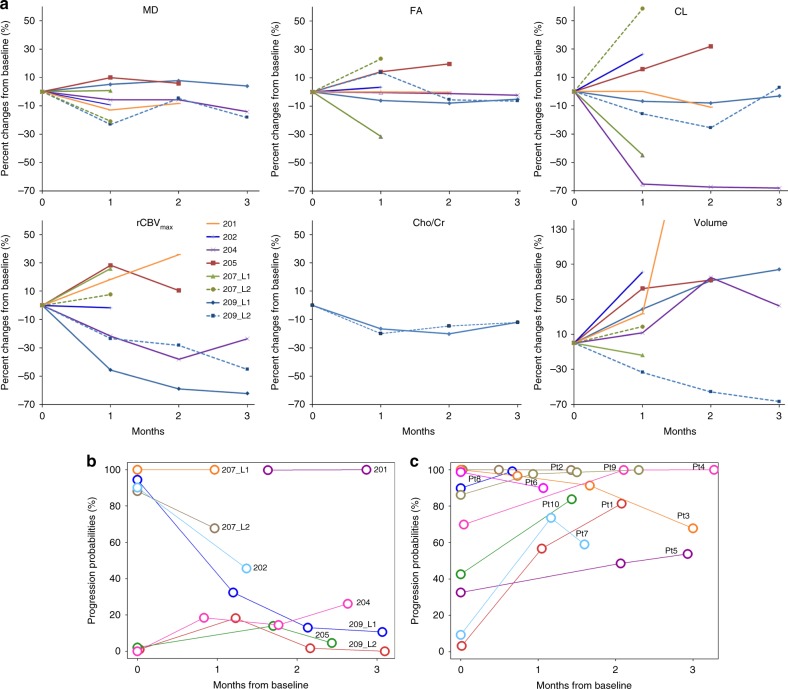
Percent changes of MR parameters and tumour volume after CAR-T treatment for eight lesions in six patients (**a**). Baseline and follow-up MRSI data was only available for two lesions to compute the percent changes. Changes of progression probabilities (PP) using the predictive model for eight lesions in six patients (**b**). Changes of PP in 10  recurrent GBM patients without CAR-T therapy (**c**). The probability of true progression is 50–100% whereas the probability of pseudo-progression is 0–50%

Serial anatomical images, parametric maps and histologic findings from patient 209 at baseline and follow-up periods are shown in Figure S1. Baseline imaging parameters and PP obtained using the classification model for the three groups are presented in Table 1. Percentage changes in tumour volume and imaging parameters at follow-up periods in comparison to baseline are shown in Fig. 1a. Six out of eight lesions demonstrated increased tumour volume at follow-up periods relative to baseline.

**Table 1:** Baseline imaging parameters and progression probabilities (PP) (mean ± standard deviation)

Parameters	Early surgery	Later surgery	No surgery
rCBV	1.84 ± 0.64 (4)	2.02 ± 0.65 (5)	1.86 ± 0.30 (3)
rCBV_max_	3.78 ± 1.14 (4)	3.95 ± 1.47 (5)	3.36 ± 0.82 (3)
MD (× 10^−3^ mm^2^/s)	1.19 ± 0.83 (4)	1.23 ± 0.23 (5)	1.41 ± 0.24 (3)
FA	0.16 ± 0.02 (4)	0.14 ± 0.05 (5)	0.13 ± 0.02 (2)
CL	0.06 ± 0.00 (4)	0.05 ± 0.03 (5)	0.08 ± 0.06 (2)
CP	0.09 ± 0.00 (4)	0.07 ± 0.02 (5)	0.07 ± 0.00 (2)
CS	0.85 ± 0.01 (4)	0.87 ± 0.04 (5)	0.83 ± 0.05 (2)
Cho/Cr	0.39 ± 0.03 (3)	0.41 ± 0.01 (2)	
PP	0.92 ± 0.13 (4)	0.57 ± 0.51 (5)	0.04 ± 0.63 (2)

The number of lesions included in each parameter are indicated in parenthesis

The enhancing lesions from four patients in “early surgery” group demonstrated high PP (72–99%) and were classified as progressive disease. These four patients underwent repeat surgery within a month following CAR-T cell infusion and were excluded from longitudinal analysis. Histopathological findings confirmed the diagnosis of TP in these patients.

For remaining six patients (total of eight lesions), three lesions (204, 205 and 209 L2) with low PP at baseline remained stable at follow-up. Two lesions (202, 209 L1) initially with high PP at baseline were associated with large decreases in PP and were classified as PsP at follow-up. The remaining two lesions (207 L1 and L2) with high PP at baseline continued to demonstrate aggressive imaging features at follow-up. No baseline data was available for patient 201. However, this patient showed features of TP at follow-up. Predicted PP values at baseline and follow-up periods are shown in Table S3. The plots of these predictive PPs are shown in Fig. 1b. Predicted PPs obtained from our classification model were confirmed on histopathology for all patients in the early and late surgery groups. Predicted PPs for recurrent GBMs without CAR-T therapy are shown in Table S4 and Fig. 1c. Eight patients showed increased PP at follow-up time points.

## Discussion

We used a predictive model from multiparametric MRI to assess the behaviour of neoplastic lesions following anti-EGFR CAR-T cell immunotherapy. All patients with available histopathology were correctly predicted as TP or PsP, indicating utility of multiparametric MRI in evaluating therapeutic response to CAR-T cell immunotherapy.

Harnessing of immune response involves inflammatory sequelae that complicates conventional MRI appearance and limits the use of RANO criteria. As immunotherapies enter clinical trials for treating GBM, there is an urgent need to reliably assess the efficacy of these treatment modalities in detecting elusive disease and redefining response.^5^

Multiparametric MRI has been widely used to predict treatment response in GBM patients.^6–8^ When percent changes in individual imaging parameters were assessed from enhancing lesions at different follow-up periods relative to baseline, no definite trends were observed, indicating that imaging parameters, in isolation, may have a limited role in assessing heterogeneity of treatment response to EGFRvIII CAR-T cell therapy. However, when we used the PP derived from multiparametric MRI, we were able to objectively characterise each lesion as either progression or response at each individual time point, suggesting that a multiparametric approach may allow more accurate characterisation of treatment response in GBM patients treated with immune/targeted therapies. These results need to be validated in a larger patient cohort and correlated with clinical endpoints of progression free survival and OS.

## Electronic supplementary material

Supplemental Material

Supplementary Table S1

Supplementary Table S2

Supplementary Table S3

Supplementary Table S4

Supplementary Figure S1
