# Supplementary material for: Multiparametric magnetic resonance imaging in the assessment of anti-EGFRvIII chimeric antigen receptor T cell therapy in patients with recurrent glioblastoma
Source: Br J Cancer. 2018 Nov 27;120(1):54–6. doi: 10.1038/s41416-018-0342-0 (PMC6325110; doi:10.1038/s41416-018-0342-0)
Supplement: Supplementary file 1 — Supplemental Material [file 41416_2018_342_MOESM1_ESM.docx]

**Methods**

Patient inclusion criteria and segmentation methods used for extracting the values from the contrast enhancing area of the tumor:

**Inclusion Criteria for selection of patients on the CART-EGFRvIII trial**

The inclusion criteria were that all patients (a) had a histologically confirmed diagnosis of GBM, (b) harbored EGFRvIII mutation, (c) had adequate organ function with Karnofsky performance status (KPS) > 60%, (d) had undergone maximal safe surgical resection followed by standard of care CCRT and adjuvant TMZ, and (e) had successful manufacturing of CART-EGFRvIII cell product.

**Tumor Segmentation**

A semi-automatic segmentation approach was used to generate a mask from the enhancing region of the tumor. Specifically, one region of interest (ROI) was manually drawn over the entire FLAIR abnormality on every slice to create a 3D composite mask. Similarly, another mask was manually drawn on the contrast-enhanced T1-weighted images for the contra-lateral normal white matter (WM). Once these ROIs were available, the enhancing region was defined as the region with enhancement higher than mean + 3SD of the signal intensity from the WM.
