# Supplementary material for: Multiparametric magnetic resonance imaging in the assessment of anti-EGFRvIII chimeric antigen receptor T cell therapy in patients with recurrent glioblastoma
Source: Br J Cancer. 2018 Nov 27;120(1):54–6. doi: 10.1038/s41416-018-0342-0 (PMC6325110; doi:10.1038/s41416-018-0342-0)
Supplement: Supplementary file 2 — Supplementary Table S1 [file 41416_2018_342_MOESM2_ESM.pdf]

Table S1. Characteristics for Patient with CAR-T therapy

	Pt	Sex	Age (y)	Location	Time from Initial Resection (d)	Line of Treat-ment	KPS	Steroid	OS from trial(d)	OS from diagnosis (d)	EGFRvIII Level (%)
Early Surgery	211	F	56	R Par	291	3	60	0	243	534	42
	213	F	52	L Par	326	3	80	DXM	284	616	70
	216	M	45	L Tem	682	3	90	0	402	1025	96
	217	M	46	L Tem	390	4	80	0	129	519	80
Late Surgery	205	F	65	L Tem	676	3	100	0	251	927	21
	207	M	77	L Tem, L Occ	462	4	80	0	186	646	95
	209	F	61	L Tem, L Occ	227	2	100	0	1033	1261	60
No Surgery	201	M	59	L BG	218	4	90	DXM	146	369	93
	202	M	75	L Tem	642	4	90	0	101	743	6
	204	F	54	R Tha	179	2	70	0	284	454	72

Abbreviation:

y years; d days; KPS karnofsky performance status; OS overall survival;

R right; L left; Tem temporal; BG basal ganglia; Occ occipital; Tha: Thalamus;

Par: Parietal; DXM Dexamethasone
