# Supplementary material for: Multiparametric magnetic resonance imaging in the assessment of anti-EGFRvIII chimeric antigen receptor T cell therapy in patients with recurrent glioblastoma
Source: Br J Cancer. 2018 Nov 27;120(1):54–6. doi: 10.1038/s41416-018-0342-0 (PMC6325110; doi:10.1038/s41416-018-0342-0)
Supplement: Supplementary file 3 — Supplementary Table S2 [file 41416_2018_342_MOESM3_ESM.pdf]

Table S2. Characteristics for Patient without CAR-T therapy

Pt	Sex	Age (y)	Location	Time from Initial Resection (d)	OS from recurre nce (d)	OS from diagnosis (d)	EGFRvIII
1	M	60	R Tem	247	352	599	Neg
2	M	45	R Tem	421	73	494	Pos
3	F	54	L Par	216	259	475	Pos
4	F	59	R Tem	649	334	983	Pos
5	F	55	R Par	171	366	537	Pos
6	M	61	L Tem	459	306	765	Pos
7	F	54	L Fro	307	329	636	Pos
8	F	63	R Fro	705	488	1193	Pos
9	M	77	L Fro	1248	227	1475	Pos
10	F	64	L Tem	721	318	1039	Pos

Abbreviation:

y years; d days; OS overall survival; Neg negative; Pos positive

R right; L left; Tem temporal; Par Parietal; Fro Frontal
