# Supplementary material for: Multiparametric magnetic resonance imaging in the assessment of anti-EGFRvIII chimeric antigen receptor T cell therapy in patients with recurrent glioblastoma
Source: Br J Cancer. 2018 Nov 27;120(1):54–6. doi: 10.1038/s41416-018-0342-0 (PMC6325110; doi:10.1038/s41416-018-0342-0)
Supplement: Supplementary file 4 — Supplementary Table S3 [file 41416_2018_342_MOESM4_ESM.docx]

**Table S3.** Predicted progression probabilities (PP %) in 12 lesions from 10 patients with CAR-T therapy using logistic regression model at baseline and follow-up time points.

|  | Pt | Baseline | 1-month | 2-month | 3-month |
| --- | --- | --- | --- | --- | --- |
| Early Surgery | 211 | 99 |  |  |  |
|  | 213 | 72 |  |  |  |
|  | 216 | 94 |  |  |  |
|  | 217 | 99 |  |  |  |
| Late Surgery | 205 | 2 | 14 | 5 |  |
|  | 207_L1 | 99 | 99 |  |  |
|  | 207_L2 | 88 | 68 |  |  |
|  | 209_L1 | 94 | 32 | 13 | 10 |
|  | 209_L2 | 1 | 18 | 2 | 1 |
| No Surgery | 201 |  | 99 | 99 |  |
|  | 202 | 90 | 45 |  |  |
|  | 204 | 17 | 18 | 14 | 26 |
