# Supplementary material for: Multiparametric magnetic resonance imaging in the assessment of anti-EGFRvIII chimeric antigen receptor T cell therapy in patients with recurrent glioblastoma
Source: Br J Cancer. 2018 Nov 27;120(1):54–6. doi: 10.1038/s41416-018-0342-0 (PMC6325110; doi:10.1038/s41416-018-0342-0)
Supplement: Supplementary file 5 — Supplementary Table S4 [file 41416_2018_342_MOESM5_ESM.docx]

**Table S4.** Predicted progression probabilities (PP %) from 10 patients without CAR-T therapy using logistic regression model at baseline and follow-up time points.

| Pt | Baseline | 1-month | 2-month | 3-month |
| --- | --- | --- | --- | --- |
| 1 | 3 | 57 | 81 |  |
| 2 | 99 | 99 | 99 |  |
| 3 | 99 | 96 | 91 | 68 |
| 4 | 70 | 99 | 99 |  |
| 5 | 33 | 49 | 54 |  |
| 6 | 98 | 90 |  |  |
| 7 | 9 | 73 | 59 |  |
| 8 | 90 | 99 |  |  |
| 9 | 86 | 97 | 98 | 99 |
| 10 | 42 | 83 |  |  |
