# Supplementary figures and images for: Multiparametric magnetic resonance imaging in the assessment of anti-EGFRvIII chimeric antigen receptor T cell therapy in patients with recurrent glioblastoma
Source: Br J Cancer. 2018 Nov 27;120(1):54–6. doi: 10.1038/s41416-018-0342-0 (PMC6325110; doi:10.1038/s41416-018-0342-0)

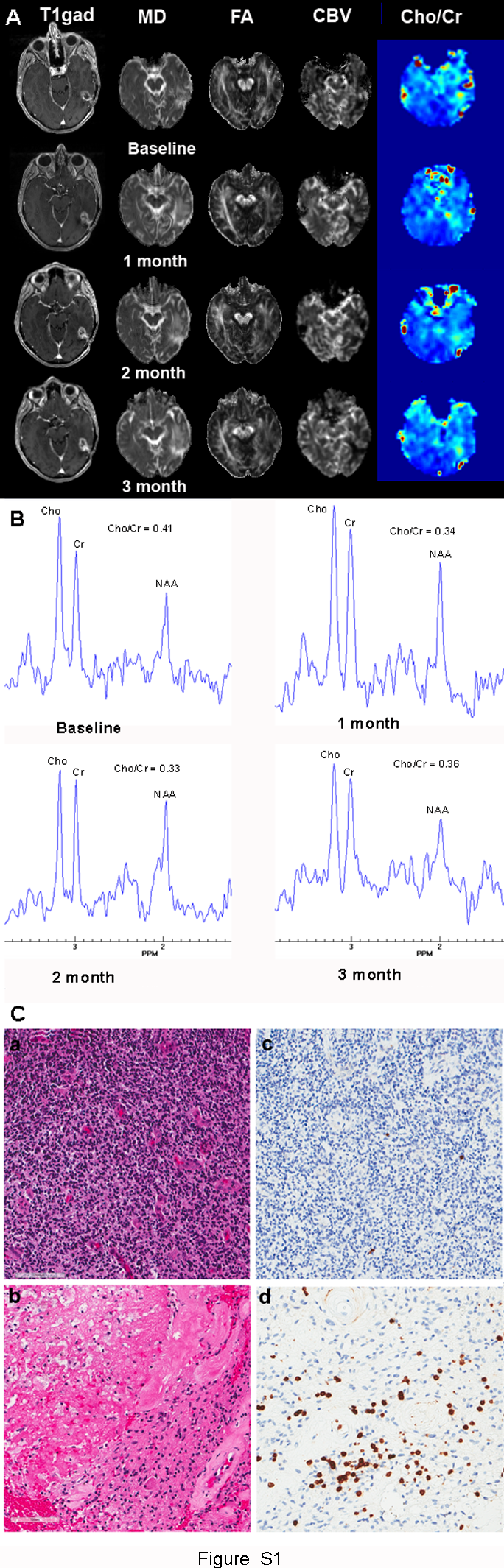

Supplement: Supplementary file 6 — Supplementary Figure S1 [file 41416_2018_342_MOESM6_ESM.tif]
